# Hysterosalpingosonography for diagnosing tubal occlusion in subfertile women: a systematic review protocol

**DOI:** 10.1186/2046-4053-2-50

**Published:** 2013-07-04

**Authors:** Sarah Maheux-Lacroix, Amélie Boutin, Lynne Moore, Marie-Ève Bergeron, Emmanuel Bujold, Philippe Y Laberge, Madeleine Lemyre, Sylvie Dodin

**Affiliations:** 1 Département d’Obstétrique, Gynécologie et Reproduction, Université Laval, 2325, Rue de l’Université, Québec, QC, G1V 0A6, Canada; 2 Centre de Recherche du CHU de Québec, 2705, Boulevard Laurier, Québec, QC, G1V 4G2, Canada

**Keywords:** Hysterosalpingosonography, HyCoSy, Hysterosalpingography, Tubal patency, Subfertility, Diagnostic test accuracy, Systematic review, Meta-analysis

## Abstract

**Background:**

Hysterosalpingosonography has been suggested as a less invasive alternative to hysterosalpingography for detecting tubal occlusion among subfertile women. We aim to determine the diagnostic accuracy of hysterosalpingosonography and to compare it to hysterosalpingography.

**Methods/design:**

We will conduct a systematic review of diagnostic test accuracy. We will search Medline, Embase, Cochrane Library, Web of Science and Biosis, as well as reference lists of included studies and previous related review articles. Diagnostic studies that compared hysterosalpingosonography ± hysterosalpingography to laparoscopy with chromotubation in women suffering from subfertility will be eligible. Two authors will independently screen for inclusion, data extraction, and quality assessment. Methodological quality will be assessed using the Quality Assessment of Diagnostic Accuracy Study 2 tool (QUADAS-2). We will use SAS 9.3 (SAS Institute Inc., Cary, NC, USA, 2011) to program bivariate random-effects models, estimate pooled sensitivity and specificity with 95% confidence intervals and to generate summary receiver operating characteristics curves. We will perform sensitivity analyses to examine the effect of differences in techniques used for hysterosalpingosonography and in methodological quality of studies.

**Discussion:**

This systematic review will help to determine if hysterosalpingosonography is an adequate alternative screening test for diagnosing tubal occlusion. Accuracy of specific sono-HSG techniques may also be identified.

**Trial registration:**

This review has been registered at PROSPERO. The registration number is CRD42013003829

## Background

Tubal factors are responsible for approximately one third of female subfertility [1]. Screening for tubal occlusion is part of the investigation of subfertile couples and is classically performed using hysterosalpingography (HSG)or laparoscopy with chromotubation [2]. Laparoscopy is largely accepted as the gold standard for diagnosing tubal occlusion [2-4]. However, being a more costly and invasive test [3], it is usually reserved for women who could also benefit from laparoscopy for the assessment or treatment of another pelvic pathology [2]. Conversely, in women without suspected comorbidities (such as pelvic inflammatory disease, previous ectopic pregnancy or endometriosis), HSG is largely accepted as a valid test for ruling out tubal occlusion [2].

More recently, hysterosalpingosonography (sono-HSG), an ultrasound-based technique, was proposed as another minimally invasive alternative to laparoscopy. Sono-HSG and HSG are both outpatient procedures that are well tolerated [5-7]. However, sono-HSG has the advantages of avoiding the use of ionizing radiation and the risk of iodine allergy [3]. Compared to HSG, sono-HSG also has greater sensitivity and specificity for detecting intrauterine pathologies [8,9] and enables concomitant visualization of the ovaries and the myometrium [3].

A previous systematic review with meta-analysis comparing sono-HSG and HSG to laparoscopy for diagnosing tubal occlusion was published in 1997 [10]. In this review, sono-HSG was associated with a 10% rate of false occlusion and 7% of false patency compared to 13% and 11%, respectively, with HSG. However, several reports have been published after this review [3]. Over the years, the techniques for sono-HSG have been improved with the arrival of new contrast media, three-dimensional (3D) ultrasonography, color-coded 3D power Doppler imaging, and increasing resolution of ultrasound [11-13].

The primary objective of our study is to determine the diagnostic accuracy of sono-HSG for detecting tubal occlusion in women suffering from subfertility. The secondary objective is to compare the diagnostic accuracy of sono-HSG and HSG in reference to laparoscopy with chromotubation. We plan to investigate sources of heterogeneity such as differences in the techniques used to perform sono-HSG and methodological quality of the included studies.

## Methods

### Design

The design of this systematic review was elaborated by a multidisciplinary group of experts (for example, reproductive endocrinology and infertility, sonography in obstetrics and gynecology, minimally invasive gynecology, research methodology) using methodological approaches outlined in the Cochrane Handbook for Systematic Review of Diagnostic Test Accuracy [14]. This protocol was written in accordance with the Preferred Reporting Items for Systematic Reviews and Meta-Analyses (PRISMA) [15] criteria and has been registered with PROSPERO (#CRD42013003829).

### Information sources and search strategy

We will search Medline, Embase, Cochrane Library, and the Web of Science from their inception. Biosis will be used to identify relevant abstracts and conference proceedings. The search strategy for Pubmed is presented in Table 1. As recommended in the literature [16-18], we developed search strategies with terms related to the index test (sono-HSG) and the target condition (tubal occlusion) and did not use any filter for diagnostic studies to maximize the sensitivity of the search strategies. All strategies were revised by a healthcare librarian and all authors. Finally, we will look at reference lists and citations of relevant articles (previous reviews and included studies) to identify any additional eligible studies.

**Table 1:** Pubmed search strategy (results from 14th November, 2012)

	**Search**	**Results**
#1	Fallopian Tube Patency Tests [Mesh] OR Hysterosalpingo-contrast-sonography [tiab] OR “Hysterosalpingo-contrast sonography” [tiab] OR “Hysterosalpingo contrast sonography” [tiab] OR Hysterosalpingo-contrast-ultrasonography [tiab] OR “Hysterosalpingo-contrast ultrasonography” [tiab] OR “Hysterosalpingo contrast ultrasonography” [tiab] OR Hysterosalpingo-foam-sonography [tiab] OR “Hysterosalpingo-foam sonography” [tiab] OR “Hysterosalpingo foam sonography” [tiab] OR Hysterosalpingosonography [tiab] OR Hysterosonosalpingography [tiab] OR Sonohysterosalpingography [tiab] OR HyCoSy [tiab] OR HyFoSy [tiab] OR Sono-HSG [tiab] OR SonoHSG [tiab] OR “Sono HSG” [tiab] OR Ultrasonography [Mesh] OR *Ultrasound* [tiab] OR *Sonogra* [tiab] OR *Ultrason* [tiab] OR *Echograph* [tiab]	387435
#2	Fallopian tube diseases [Mesh] OR ((Fallopian tubes [Mesh] OR Tubal [tiab] OR tube [tiab] OR tubes [tiab]) AND (patenc* [tiab] OR block* [tiab] OR occlusi* [tiab] OR obstructi* [tiab] OR damage* [tiab])	15021
#3	Animals [Mesh] NOT Humans [Mesh]	3728027
#4	#1 AND #2 AND #3	962

### Eligibility criteria and study selection

We will consider all studies assessing the diagnostic accuracy of sono-HSG for diagnosing tubal occlusion among a subfertile population. There will be no restriction in terms of publication date or language. Studies published in languages other than English or French will be translated. Studies including women suffering from recurrent spontaneous miscarriages will be eligible. However, we will exclude studies that include populations having no desire for fertility (for example, assessment of tubal occlusion after a hysteroscopic tubal occlusion procedure).

Laparoscopy with chromotubation is widely recognized as the gold standard in assessing tubal patency [2,3] as its findings are highly correlated with spontaneous pregnancy rate [4]. Thus, we will consider exclusively studies using laparoscopy as reference standard. Studies considering other modalities as gold standard, such as HSG, hysteroscopic selective tubal cannulation under fluoroscopic guidance or vaginal laparoscopy, will be excluded [19]. For studies using HSG as a comparator test (that is, additionally assessing the accuracy of HSG compared to laparoscopy), data on the diagnostic accuracy of HSG will be retained in order to make a direct comparison of accuracy between sono-HSG and HSG.

We will consider consecutive and random series of patients as well as case-control designs. For studies using partial verification (gold standard achieved only in a subgroup of participants), we proceeded according to recent recommendations [20,21]. Studies with partial verification will not be systematically excluded in order to obtain an increased precision and a better generalizability. Studies using a random partial verification will be included as they are not prone to the partial verification bias. In cases of non-random partial verification, studies will be included in the review only if adjustment for the verification bias is possible. That is, if determinants of the partial verification are known and verification in each strata is random and in known proportions.

Eligibility assessment will be performed independently by two reviewers screening titles, abstracts, and full text publications, when required. If disagreements are not resolved by consensus, a third reviewer will be consulted. We will collect reasons for full-text exclusion. To avoid duplication, author names, sample sizes and results of studies will be compared.

### Data collection

Two reviewers will extract data from included studies and disagreements will be resolved by discussion. If consensus is not reached, a third reviewer will be consulted. We developed a standardized data abstraction form, which was pilot-tested on three studies [22-24] and refined accordingly. The following information will be extracted from each study:

1) study characteristics and methods: study design, inclusion and exclusion criteria, flow diagram, setting, country, language of publication;

2) description of the technique used for sono-HSG: resolution, two-dimensional (2D) or three-dimensional (3D) device, vaginal or abdominal probe, type of contrast, type of catheter, and use of Doppler;

3) measures of diagnostic accuracy of sono-HSG (and HSG when available) in reference to laparoscopy.

For comparison purposes, we will consider a non-patent tube as a positive test and data of studies will be converted if needed. If published data does not allow us to obtain or derive the number of true positives (TP), false positives (FP), true negatives (TN) and false negatives (FN), we will attempt to contact the corresponding author of the study.

### Assessment of methodological quality

Two reviewers will independently assess the risk of bias and applicability concerns using a checklist derived from the *Quality Assessment of Diagnostic Accuracy Study 2 (QUADAS-2) tool*[25] (web appendix). In case of discrepancy, a third reviewer will be consulted. An interval of no more than one month between the tests will be considered appropriate. Sono-HSG (± HSG) results must be interpreted without the knowledge of the results of laparoscopy. However, we will consider the lack of blinding in the interpretation of laparoscopy results to be associated with a low potential for bias. Reviewers’ judgments about risks of bias and applicability concerns will be used in sensitivity analyses to examine the effects of methodological quality of studies.

### Outcomes

The primary outcome will be the diagnostic accuracy of sono-HSG for detecting tubal occlusion. The secondary outcome will be the direct comparison of the diagnostic accuracy of sono-HSG and HSG.

### Statistical analysis and data synthesis

Meta-analyses will be performed by computing TP, TN, FP, and FN results of each study in bivariate hierarchical random-effects models using SAS 9.3 (SAS Institute Inc., Cary, NC, USA, 2011). Cochrane Review Manager version 5.2 (The Nordic Cochrane Centre, The Cochrane Collaboration, Copenhagen, Denmark, 2012) will be used to present the results. Pooled and individual estimates of sensitivity and specificity and 95% confidence intervals (CI) will be presented in forest plots. We will generate summary receiver operating characteristics (SROC) curves using point estimates for each study as well as a symmetrical summary curve, a summary point estimate, 95% confidence region, and 95% prediction region. Direct comparison of sono-HSG and HSG accuracy will also be achieved using bivariate models.

The magnitude of heterogeneity will be assessed by calculating the 95% prediction region on SROC curves [26]. We will perform sensitivity analyses with bivariate hierarchical random-effects model and examine the effect of differences in the technique used for sono-HSG (2D versus 3D, low (≤ 5 MHz) versus high resolution (> 5 MHz), use of Doppler, vaginal versus abdominal probe, Echovist versus saline, and use of a catheter with balloon versus catheter without balloon), and in methodological quality of studies (low versus high or unclear risks of bias and applicability concerns).

## Discussion

Assessment of tubal patency is an important part of the investigation of infertile couples as tubal factors are responsible for almost one third of cases of female subfertility [1]. The proposed systematic review is based on recommended methodological approaches [14,15,25] and will determine the accuracy of sono-HSG for the diagnosis of tubal occlusion in women suffering from subfertility. This review will also permit comparison of sono-HSG with HSG, the latter being widely used and accepted as a reliable test for ruling out tubal occlusion in this population [2,3,27,28].

Previous diagnostic test studies conducted on the subject have reported variable results, which may be linked to imprecision or variable methodological quality. Discrepancies may also be associated with differences in the techniques used for sono-HSG, which have greatly changed in the past decade with technological developments [11-13]. This systematic review will allow us to determine whether sono-HSG is an adequate screening test for diagnosing tubal occlusion. It will also determine if sono-HSG could replace HSG for the screening of tubal occlusion considering that sono-HSG is associated with several advantages over HSG including better diagnostic accuracy for uterine pathologies [8,9], concomitant assessment of ovaries and myometrium and avoidance of radiation and risk of iodine allergy [3]. The systematic review could also identify specific sono-HSG techniques associated with greater sensitivity and/or specificity. We plan to communicate the results of the review by presenting research abstracts at conferences and by publishing the results in a peer-reviewed journal.

## Web appendix

### Quality checklist derived from QUADAS-2 tool

1. Patient selection

a. *Risk of bias*

•••Was a consecutive or random sample of patients enrolled? *Yes, No, Unclear*

•••Was a case-control design avoided? *Yes, No, Unclear*

•••Did the study avoid inappropriate exclusion? *Yes, No, Unclear*

•••Could the selection of patients have introduced bias? *Low, High, Unclear*

a. *Concern regarding applicability*

•••Is there concern that the included patients do not match the review question? *Low, High, Unclear*

2. Index tests: hysterosalpingosonography (sono-HSG) ± hysterosalpingography (HSG)

a. *Risk of bias*

•••Was the sono-HSG ± HSG results interpreted without knowledge of the results of the reference standard? *Yes, No, Unclear*

•••Was the definition of a positive test pre-specified? *Yes, No, Unclear*

•••Could the conduct or interpretation of the sono-HSG ± HSG have introduced bias? Low, High, Unclear

a. *Concern regarding applicability*

•••Is there concern that sono-HSG ± HSG, its conduct, or interpretation differs from the review question? *Low, High, Unclear*

3. Reference standard: laparoscopy with chromotubation

a. *Risk of bias*

•••Is there a standard likely to correctly classify the target condition? *Yes, No, Unclear*

•••Could the conduct or interpretation of the laparoscopy have introduced bias? *Low, High, Unclear*

a. *Concern regarding applicability*

•••Is there concern that tubal patency as defined by the reference standard does not match the review question? *Low, High, Unclear*

4. Flow and timing

a. *Risk of bias*

•••Was there an interval of no more than one month or one cycle between the index test(s) and the reference standard? *Yes, No, Unclear*

•••Did patients receive the same reference standard? *Yes, No, Unclear*

•••Did all patients in the analysis receive the reference standard? *Yes, No, Unclear*

•••Were at least 90% of eligible patients included in the analysis? *Yes, No, Unclear*

•••Could the patient flow have introduced bias? *Low, High, Unclear*

## Abbreviations

2D: Two-dimensional; 3D: Three-dimensional; CI: Confidence interval; FN: False negative; FP: False positive; HSG: Hysterosalpingography; QUADAS-2: Quality Assessment of Diagnostic Accuracy Study 2; TN: True negative; TP: True positive; Sono-HSG: Hysterosalpingosonography; SROC: Summary receiver operating characteristic.

## Competing interests

The authors declare that they have no competing interests.

## Authors’ contributions

SML, SD, MEB, PL and ML developed the objectives of the review and established its relevance in relation to the current literature. SML and AB developed the search strategies and the data abstraction form, which were then approved by the rest of the authors. SML, AB, LN, SD and EB ensured the methodological quality of the protocol. SML drafted the first version of the manuscript with significant input from all other authors. All authors have read and approved the final version of the manuscript.

## Authors’ information

SML is a resident in obstetrics and gynecology and PhD candidate in epidemiology at Université Laval. AB is a PhD candidate in epidemiology at Université Laval and specializes in methodology related to systematics reviews. LM is Assistant Professor at Université Laval and biostatistician and epidemiologist at the Centre de Recherche du CHU de Québec. MEB is a gynecologist who performed a fellowship in reproductive endocrinology and infertility at the University of Oxford. EB is Associate Professor in Obstetrics and Gynecology at Université Laval and clinician-scientist at the Centre de Recherche du CHU de Québec with expertise in obstetrical and gynecological sonography and in systematic reviews. PL and ML are gynecologists specialized in minimally invasive gynecology at the CHU de Québec. SD is Professor in Obstetrics and Gynecology at Université Laval and clinician-scientist at the CHU de Québec, with expertise in systematic reviews.
